# Syndemics in women’s health: poverty, social exclusion, and clustering of thrombotic and hemostasis disorders

**DOI:** 10.1016/j.rpth.2024.102481

**Published:** 2024-06-20

**Authors:** Ellen O’Rourke, Sarah Kelliher, Barry Kevane

**Affiliations:** 1 Haematology, Mater Misericordiae University Hospital, Dublin, Ireland; 2 School of Medicine, University College Dublin, Dublin, Ireland; 3 Irish Network for Venous Thromboembolism Research, Dublin, Ireland

**Keywords:** hemorrhage, poverty, social determinants of health, syndemic, thrombosis

## Abstract

A State of the Art lecture titled “Syndemics in Women’s Health: Poverty, Social Exclusion and Clustering of Thrombotic and Haemostasis Disorders” was presented at the International Society on Thrombosis and Haemostasis (ISTH) Congress in 2023. Syndemics are characterized by the clustering of specific health conditions in vulnerable populations. These populations become vulnerable as a result of large-scale social, political, and economic factors that influence social determinants of health and increase susceptibility to disease. Vulnerable populations at risk of experiencing a syndemic include those who are subjected to social exclusion and gender- or race-based marginalization. Biological sex (assigned at birth based on physical & genetic differences) and gender identity (the personal sense of ones own gender) have been recognized as important determinants of health outcomes in the context of certain syndemic diseases. Potential examples of syndemic biosocial interactions in the field of thrombosis and hemostasis include the effect of social determinants of health in perpetuating the global maternal mortality crisis and the role of poverty and marginalization in influencing thrombosis risk in socially excluded individuals. Initiatives directed at prevention and treatment of syndemic conditions require multilevel interventions directed at the socio-economic as well as the biological determinants of the disease. In the present article, we describe potential syndemic disease interactions in the field of thrombosis and hemostasis, and we summarize some relevant new data relating to the social determinants of health presented during the 2023 ISTH Congress.

## Essentials

• Syndemics are clusters of health problems driven by social, political, and economic forces.
• Syndemics of infectious and cardiovascular diseases are among the most widely described to date.
• Economic and gender-based marginalization increases vulnerability to syndemic conditions.
• Health risks relating to thrombosis and bleeding are influenced by social determinants of health.

## 1 Introduction

Health and illness are not equally distributed across society. Poor health disproportionately affects poor people, and the increased vulnerability to disease, which emerges as a consequence of poverty, is frequently compounded by inequitable distribution of healthcare resources [1,2]. The effects of poverty on health outcomes are often enduring and may even be life-long [[3], [4], [5]]. Socio-economic deprivation can have long-lasting health consequences even when arising during the *in utero* and early childhood stages of life, where it can be associated with increased risks of neurocognitive, inflammatory, and cardiovascular disease in adulthood [[6], [7], [8], [9], [10]]. The 2010 UK-based *Fair Society* *Healthy Lives Report* (*the Marmot Review*) [3] and the subsequent *Health Equity in England Report* in 2020 [4] showed that people living in the poorest neighborhoods spend more of their lives experiencing ill health (with obesity, cardiovascular disease, and diabetes mellitus all following a clear socio-economic gradient) and die on average 7 years earlier than counterparts living in richest neighborhoods [3,4].

The effects of poverty on health outcomes may be conceptualized through the social determinants of health framework [11]. These determinants relate to our ability to access healthcare, housing, nutrition, and education and are influenced by the large-scale social, political, and economic conditions in which we are born, grow, work, live, and age [11]. Inequities in the social determinants of health must be addressed when designing interventions directed at the prevention or treatment of diseases that are rooted in socio-economic marginalization [12]. Focusing entirely on individual-level health behaviors or health choices in that setting would fail to address the overarching social, cultural, and economic factors that perpetuate intergenerational cycles of ill health in the first instance [13].

In public health medicine, syndemic theory has emerged as a means of understanding why certain health conditions cluster in marginalized groups and how these conditions interact with each other and the broader social context to worsen health outcomes [1,2,[14], [15], [16], [17], [18], [19]]. Syndemic theory provides a framework to identify and design multilevel interventions that aim to improve the health of vulnerable populations, recognizing that factors such as poverty, homelessness, and gender- or race-based marginalization exert profound effects on the ability to live a healthy life [2,12,13]. It is noteworthy that both biological sex and gender identity have been identified as important factors influencing susceptibility to ill health in syndemics. Female sex is associated with increased vulnerability to ill health in the setting of socio-economic adversity, particularly when coupled with other forms of social, political, or cultural marginalization [[19], [20], [21], [22], [23], [24]]. Nonconforming gender identity/nonheterosexuality may also influence health outcomes among those who are already subjected to socio-economic and cultural marginalization [[25], [26], [27], [28]]. In this article, we describe the origins of syndemic theory and its early application in the field of infectious diseases. We also outline how certain biosocial factors and health risks relating to disorders of thrombosis and hemostasis may reflect potential emerging syndemics. In particular, we discuss the role of gender-specific social factors in influencing postpartum hemorrhage (PPH) risk (the leading global cause of maternal mortality) and the associations between social, economic, and cultural factors and the risk of thrombosis as examples of potential syndemics. Finally, we will also describe recent data presented at the ISTH 2023 congress relating to the importance of socio-economic factors in influencing disease risk and as future targets for intervention.

## 2 Syndemic Theory: Origins and Potential Applications in the Field of Thrombosis and Hemostasis

**Figure 1:**
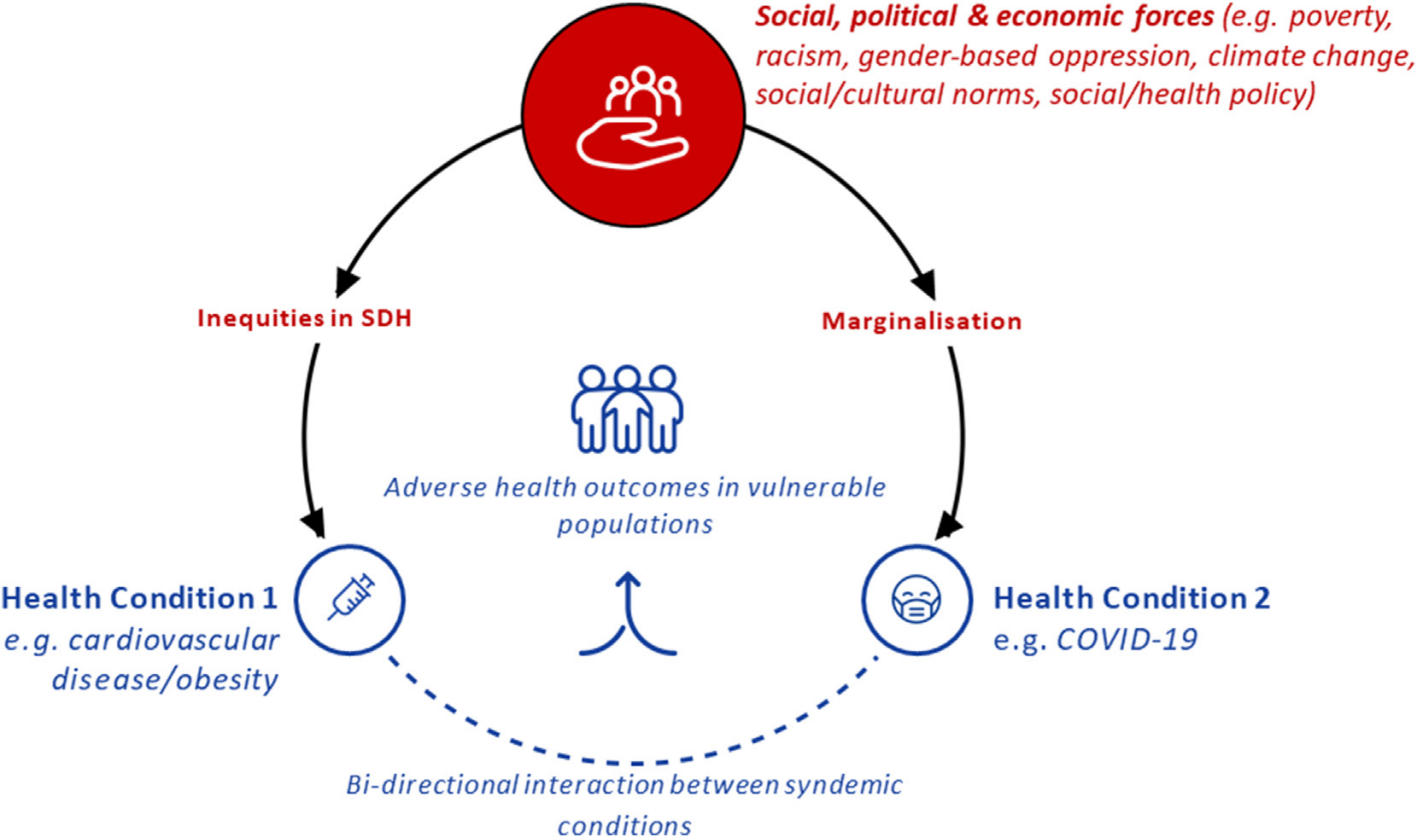
The core features of a syndemic. A syndemic emerges when 2 or more health conditions arise in a population made vulnerable by large-scale social, political, and economic factors. These external factors, such as racism or gender-based oppression, drive inequities in social determinants of health (SDH) and lead to marginalization of certain groups. Susceptibility to disease is increased, and biosocial interactions between the emerging health conditions further exacerbate the overall burden of ill health (eg, bidirectional interaction between diseases that are increased in low socio-economic status groups, such as cardiovascular disease/obesity and COVID-19; figure created with Biorender.com).

Syndemics are characterized by the clustering of multiple interacting health conditions in vulnerable populations (Figure 1) [13,29]. The 3 core features of a syndemic are 1) the clustering of 2 or more health conditions within a population, 2) where that population has been made vulnerable to these health conditions by large-scale social/political/economic factors, and 3) where these health conditions interact with each other and the broader social context to lead to an overall worsening of health outcomes [13,30,31]. Syndemic theory emerged in the 1990s in response to the disproportionate burden of HIV/AIDS, which was being observed among specific disadvantaged minority groups in the United States despite advances in HIV/AIDS awareness and care at the time [27]. This ***s****ubstance* ***a****buse,* ***v****iolence, and* ***A****IDS* (SAVA) syndemic was the first instance in which syndemic theory was used to understand the origins of a public health emergency. In the description of the SAVA syndemic, it was proposed that the decades of structural racism and socio-economic deprivation, which had fueled poverty, housing instability, substance misuse, and drug-related violence in poor, urban, and predominantly Black communities, had led to increased risks of HIV transmission [27]. Moreover, the intersection between these health conditions, the prevailing structural barriers, and the social context in which they were rooted negatively impacted the health behaviors of affected individuals and their ability to access healthcare resources. Using the syndemic model, upstream contextual determinants of disease can be identified and addressed [13]. For example, housing instability greatly amplified vulnerability to a multitude of health conditions and was recognized as a causative factor (and target for intervention) in the SAVA syndemic [[32], [33], [34]].

While disorders of blood coagulation have yet to be extensively studied within the context of the syndemic framework, there are a number of conditions linked to the field of thrombosis and hemostasis that exhibit clear associations with socio-economic status (SES) and which may be amenable to syndemic intervention in the design and delivery of health care services.

Low-income females (including biological females and noncisgender people who identify as women) appear to exhibit particular vulnerability to the adverse effects of syndemic interactions, largely due to the specific socio-political-economic factors that tend to marginalize these groups [19,[22], [23], [24]]. This may also represent an important factor in hematology when considering the socio-economic roots of the global maternal mortality crisis (and specifically with regard to PPH), as well as the thrombotic and vascular risks experienced by socially excluded women and gender minorities.

### 2.1 Syndemics, PPH, and the global maternal mortality crisis

The World Health Organization estimated that 287,000 maternal deaths occurred in 2020 alone, a rate which is equivalent to approximately 800 maternal deaths per day [35]. The vast majority of these maternal deaths are thought to have been preventable. Moreover, the vast majority of the women who died lived in low-income countries, with over 70% from sub-Saharan Africa. In this region, in 2020, a 15-year-old girl would be predicted to have a lifetime risk of death during pregnancy or childbirth of approximately 1 in 40 [36]. This figure is 400-fold higher than the lifetime maternal mortality risk in the middle-to-high-income nations where the lowest rates of maternal deaths are recorded. The United Nations Population Fund has described this difference in maternal death rates as a marker that demonstrates discrepancies in health outcomes between rich and poor nations better than any other single measure of population health [36].

PPH represents the single largest source of preventable maternal morbidity and mortality in low-income countries [37]. It has been suggested that social and cultural factors may exert an equal, if not greater, influence than biological determinants on the maternal mortality risk in these regions [36,38]. In these populations, increased vulnerability to poor health can arise as a consequence of gender-based oppression and the social or cultural norms that cause female health issues to be deprioritized. The removal of autonomy relating to decision-making on unintended pregnancy management and contraceptive use is a clear upstream determinant of maternal morbidity and mortality risk/PPH (it has been estimated that more than 200 million women in the developing world have an unmet need for contraception) [39]. Similarly, level of education and literacy are recognized as major determinants of maternal mortality risk among low-income women, particularly in sub-Saharan Africa. Here, it has been estimated that the risk of maternal death is 3-times higher among women with no formal education in contrast to those who have completed secondary-level education (and who are better equipped to seek out healthcare services and are more empowered to make their own healthcare decisions) [36,38].

**Figure 2:**
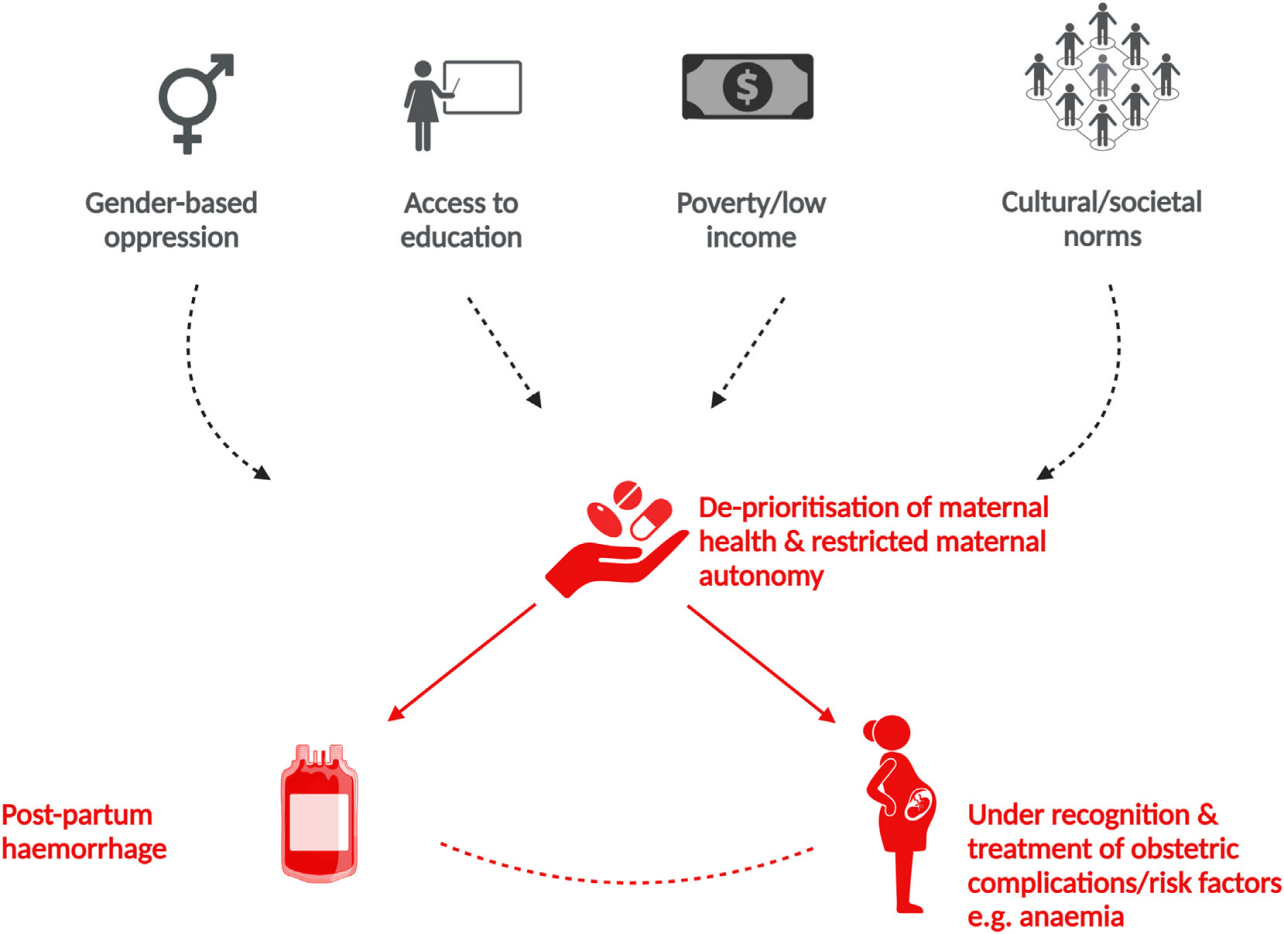
Social determinants of maternal mortality and syndemic risk factors. In low-income countries, social determinants of maternal health and postpartum hemorrhage risk include the level of education provided to women and girls, level of household income, and the effects of gender-based oppression in influencing autonomy over reproductive health choices. Deprioritization of maternal health issues, underrecognition/treatment of associated conditions such as maternal anemia, and structural barriers to accessing obstetric care increase the morbidity risk associated with postpartum hemorrhage (figure created with Biorender.com).

The effects of poverty, gender inequality, and barriers to education in making females vulnerable to adverse pregnancy outcomes, such as morbidity/mortality due to PPH, appear to reflect the core elements of a syndemic (Figure 2). Biological determinants of PPH risk are, of course, important targets for intervention, and the recent clinical trial data demonstrated that the early administration of tranexamic acid substantially reduces the risk of death from PPH and has changed practice globally [40]. However, notwithstanding this landmark advance in maternal care, the effect of interacting social and cultural factors on the biology of PPH risk must not be ignored as deaths from PPH remain unacceptably high in low-income countries where delayed access to care for women and underrecognition/undertreatment of associated health problems such as maternal anemia are frequently implicated as contributory factors in PPH mortality [41]. The deprioritization of maternal health issues and the deep-seated restrictions affecting the autonomy of females in healthcare decision-making need to be recognized as central targets for “upstream” intervention. Consequently, advocacy for improved access to education and healthcare as social determinants of the health of females remains a priority of the United Nations Population Fund and World Health Organization.

### 2.2 Poverty, social exclusion, and venous thromboembolism: a syndemic in the making?

Low SES appears to be associated with an increased risk of venous thromboembolism (VTE). In a large Danish registry study incorporating data from more than 50,000 individuals with prior VTE, matched against over 250,000 controls in the general population, the incident rate of VTE was lower among individuals who had achieved higher levels of SES [42]. This was observed for each individual indicator of SES selected by the investigators, including level of education (odds ratio [OR], 0.74; 95% CI, 0.71-0.77), level of income (OR, 0.7; 95% CI, 0.68-0.72), and employment status (OR, 0.66; 95% CI, 0.64-0.68) and for the composite of all 3 indicators of SES (OR, 0.61; 95% CI, 0.59-0.63) when comparing low against high SES groups [42]. Other investigators have reported similar findings in large nationwide/population-level studies. Isma et al. [43] examined VTE incidence in Sweden in a nationwide epidemiologic study, which demonstrated associations between levels of disposable income and VTE incidence (higher rates of incident VTE among individuals in lower to middle tertiles for household income). Neighborhood SES has also been shown to influence VTE risk (with lower VTE rates reported for individuals living in neighborhoods with highest SES), and lower rates of VTE have also been reported as being associated with higher-status occupations [44,45].

The potential mechanisms that may underlie this association between VTE risk and SES remain to be fully elucidated. However, very clear associations between socio-economic factors and cardiovascular disease in general have been reported [8]. The social determinants of vascular disease include health behaviors/health choices, which are, in turn, driven by socio-economic factors. The ability to access health care services/screening for vascular risk factors, the ability to afford a healthy diet, and the likelihood of being able to prioritize healthy living when living in poverty influence the risk of cardiovascular disease in poorer communities [8]. This has previously been described in the ***V****iolence,* ***I****mmigration,* ***D****epression,* ***D****iabetes, and* ***A****buse* syndemic, which was characterized by the synergistic adverse health effects of cardiometabolic disease and psychological morbidity in marginalized female Mexican immigrants in the United States [19]. Recently, the interplay between severe deprivation, psychological morbidity, and cardiovascular events has become a major focus of clinical and scientific research [46]. The “allostatic load” refers to the cumulative physiological damage associated with prolonged and severe psychological stress and deprivation [47]. These effects appear to be mediated through stress-related neurobiological responses in the hypothalamic-pituitary-adrenal axis [46]. Although the precise pathophysiological mechanisms are likely to be heterogeneous and have yet to be fully elucidated, it appears that the adverse effects of heightened “allostatic load” are characterized by abnormal stress hormone signaling leading to altered metabolic responses, increased hematopoietic tissue activity, and vascular/arterial inflammation; these responses can then culminate in an increased risk of major adverse cardiac events [46,47]. Elements of this phenomenon in the setting of social deprivation have been reported by numerous health researchers across multiple disciplines of medicine, demonstrating associations between socio-economic deprivation (particularly in childhood/early adulthood) with either increased clustering of cardiac diseases or elevated inflammatory/cardiometabolic markers in later life [8,10,46,48,49]. The degree to which these mechanisms might potentially be extrapolated to the field of venous thrombosis remains to be determined and at present is speculative. However, the interplay between inflammation and thrombosis risk, in general, is increasingly recognized as a potential source of morbidity, and so this may warrant additional investigation in the setting of poverty and socio-economic deprivation.

In line with the syndemic model, further biosocial interactions leading to adverse outcomes after a VTE diagnosis have been observed in studies that have demonstrated differences in the approach of healthcare providers to the management of VTE among individuals from different ethnic groups and socio-economic strata. Outcomes from a large US-based retrospective study incorporating data from Medicare and National Inpatient Sample databases suggested that individuals from ethnic minorities and nonprivately insured patients were less likely to receive advanced therapies in the setting of high-risk pulmonary embolism (PE) [50]. Other studies have suggested that older hospitalized patients from low SES groups were also less likely to be offered advanced therapies for PE and that individuals from lower SES groups were less likely to be offered specialist follow-up [51,52]. Differences in clinical outcomes have also been reported, with data suggesting that non-Caucasian ethnicity and low SES are associated with worse all-cause mortality in low SES groups [50,[53], [54], [55]]. Among survivors of VTE, an increased burden of chronic morbidity has been reported. While chronic disability following acute VTE occurs to some extent across all strata of SES, much higher rates of permanent disability and exclusion from the labor market have been reported among individuals with low SES who experience VTE [56,57]. This observation may reflect differences that may exist in the types of occupations that are more common in low-income groups (eg, occupations requiring manual labor, which may be challenging in those with chronic physical morbidity post-VTE). The implications of permanent work-related disability in this setting are significant, given the potential for this to further exacerbate socio-economic adversity.

The potential to observe a syndemic related to VTE and SES is perhaps heightened at extremes of socio-economic adversity. In middle-to-high-income countries, “social exclusion” represents perhaps the most severe state of marginalization and deprivation [34,58]. Social exclusion has been defined as a complex multidimensional state characterized by the inability of individuals to participate in the normal social, economic, and political activities of life as a result of extreme marginalization [32]. The term is generally used to refer to the lived experiences of several overlapping groups, including people experiencing homelessness, people who are incarcerated, people with substance misuse disorders, sex workers, and certain ethnic minority groups [58,59]. The overarching biopsychosocial drivers of social exclusion tend to overlap across these populations, which are frequently affected by poverty, unemployment, family breakdown, and housing instability. Healthcare services are not designed to meet the complex care needs of socially excluded people who, consequently, have extremely high rates of early morbidity and mortality (frequently driven by high rates of mental health and cardiovascular disease) [32,60]. The increased vulnerability to the health effects of social exclusion appears to be particularly striking among females [32].

Injecting drug use (IDU) is highly prevalent among socially excluded individuals and, in particular, among people experiencing homelessness. Opioids, such as heroin and crack cocaine, are the most commonly injected substances. Over 50% of homeless people who engage in IDU eventually resort to injecting into the groin/femoral veins as a source of venous access [61]. Groin injecting is a major risk factor for VTE, with a recent systematic review and meta-analysis estimating the prevalence of deep vein thrombosis (DVT) among people who inject drugs (PWID) at almost 30% [62]. DVT and related complications are a leading cause of hospital admission among PWID [63]. The mechanisms underlying VTE risk associated with IDU are numerous but primarily relate to repetitive vascular injury, unsanitary injecting practices and infection, and the direct toxic effect of the injected substances, particularly in the case of injecting crack cocaine, which appears to have a particular irritant effect on the vascular wall [64,65]. Several investigators have reported that the risk of VTE associated with IDU is higher among women and older PWID for reasons that remain unclear. It has been postulated that this may relate to patterns of drug use (increased use of stimulants such as crack cocaine as opposed to opioids, increased use of the groin as an injecting site, etc.); however, this remains to be clarified [[64], [65], [66]]. One report describing patterns of DVT presentations in 2 large urban centers in Scotland detailed that over 50% of cases of isolated DVT among women under 40 years of age attending their centers were related to IDU [67].

**Figure 3:**
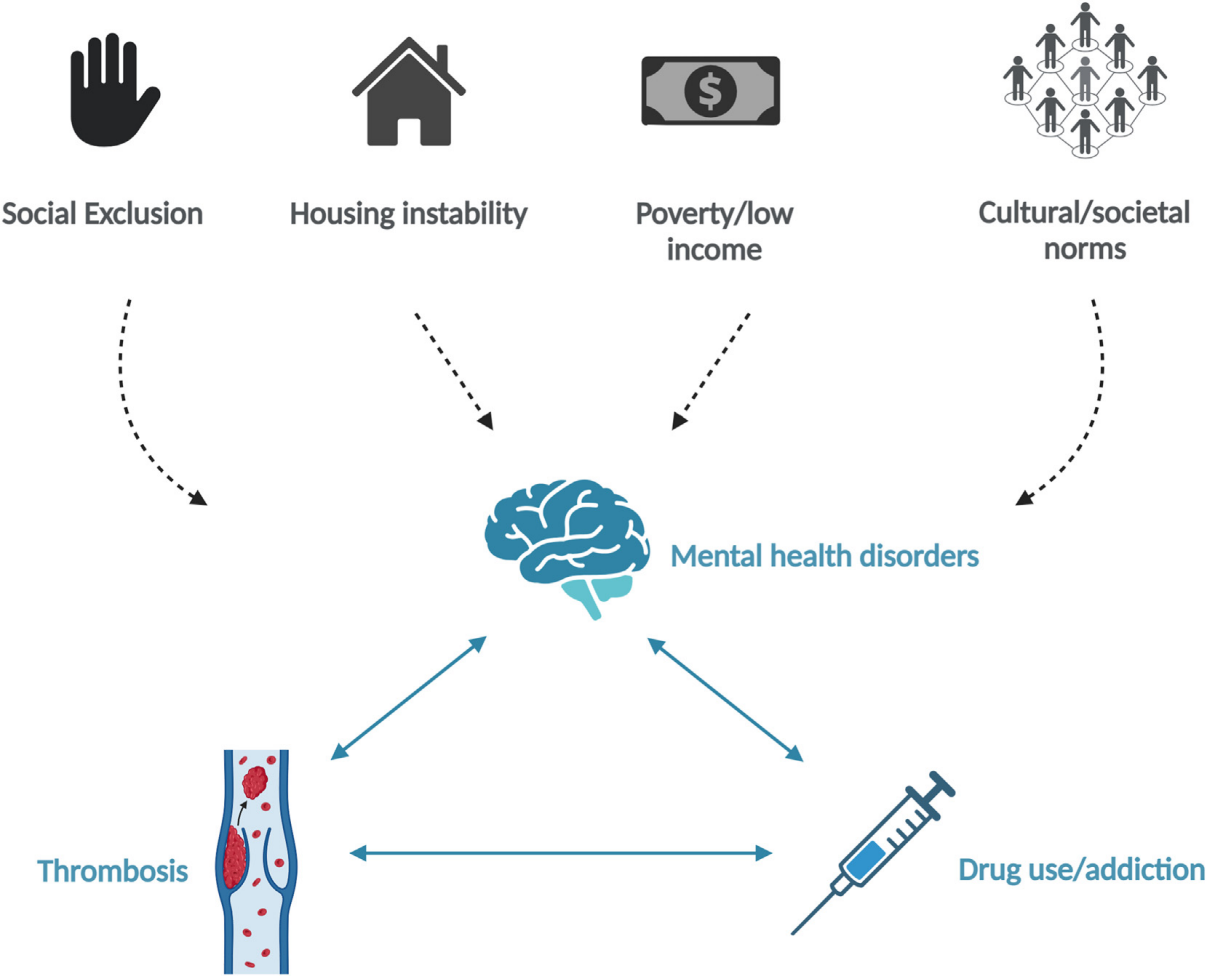
Poverty, social exclusion, and thrombosis—a syndemic in the making? Socio-economic drivers of mental health disorders and drug misuse include social exclusion, poverty, and housing instability. Thrombosis risk emerges as an important comorbidity in this setting. Its management and complications are influenced by structural and social barriers faced by people who experience housing instability and addiction, leading to suboptimal healthcare delivery and potentially further adverse health consequences (figure created with Biorender.com).

The co-occurrence of IDU, mental health conditions, and HIV have already been the subject of studies of syndemic disease interactions. These data demonstrate evidence of clustering of these conditions among women (particularly women from ethnic/racial minorities) who have been exposed to (and made vulnerable by) poverty and adverse life events (including gender-based violence, childhood trauma, and economic deprivation) [[22], [23], [24],^68^,^69^]. Thrombosis as a complication of social exclusion has yet to be described in the context of a syndemic per se, but thrombosis/vascular risk arises as a consequence of the same broader socio-economic drivers (including poverty and substance misuse) in this setting and emerges within these vulnerable populations alongside mental health conditions, homelessness, and addiction [14,62] (Figure 3). In Ireland, we have recently reported national-level data demonstrating an excess of concurrent VTE among hospitalized individuals identified as socially excluded using a composite variable incorporating interacting health/social conditions such as homelessness, drug misuse, and hepatitis C [70].

The potential for multiple syndemic biosocial interactions between co-occurring health conditions is perhaps greater among females in the context of socio-economic deprivation and thrombosis risk [71,72]. With respect to reproductive health and its implications for thrombosis risk and management, it is noteworthy that data suggest that up to 13% of homeless females may be pregnant at any 1 time, a rate which is significantly higher than the general female population [73]. The majority of these pregnancies are unintended, but the provision of contraceptives to socially excluded females represents a major unmet need, with significant structural barriers (such as inadequate counseling, cost, etc.) limiting access [74]. Menstruating people who are treated with anticoagulants are at increased risk of abnormal uterine bleeding [75]. In the setting of poverty and social exclusion, the implications of this may be exacerbated by “period poverty” and difficulties accessing menstrual hygiene products and treatment [76]. The socio-economic implications of this issue cannot be underestimated when considering the potential interaction between the complications of abnormal uterine bleeding, its effect on the health and well-being of the affected person, and their ability to work or engage in education/training in the setting of concurrent socio-economic adversity/social exclusion [77]. Transgender people are also overrepresented within the population of people experiencing homelessness. Social, cultural, and economic factors increase the vulnerability of transgender people to become homeless and drive the high prevalence of mental health conditions and IDU in this subgroup [25,26]. Trans men encounter specific challenges in managing menstrual health and the complications of abnormal uterine bleeding in the setting of social exclusion. Among trans women, access to vital gender-affirming hormonal therapy may be difficult in the setting of homelessness/severe poverty and is frequently obtained illicitly and without medical supervision, which, of course, may further add to the associated thrombosis risk [25,78].

### 2.3 A potential syndemic of poverty, thrombosis, and social exclusion: implications for health care provision?

A key purpose of applying a syndemic framework when studying the origins of disease is to drive the identification of multilevel interventions to improve health, which do not focus solely on attributing disease risk to individual-level risk behaviors [13]. Syndemic theory recognizes that marginalized people are frequently consigned to a lifetime of poor health by the large-scale social, political, and economic forces that affect the circumstances of their daily lives.

Current healthcare service models rarely meet the needs of individuals experiencing social exclusion [34,58]. Within our institution in inner city Dublin, which serves a diverse urban population, up to 20% of new referrals to the thrombosis clinic relate to individuals experiencing social exclusion (primarily PWID and people experiencing homelessness) [79]. The provision of care is challenging, with high rates of nonattendance and loss of follow-up. These challenges may be in part related to the effects of the cognitive burden of poverty, which is a concept that refers to the effect of the daily experience of severe deprivation on an individual’s pattern of cognition, decision-making, and prioritization of health needs [80]. Other important barriers to accessing care in this setting include issues relating to literacy, geographic location, access to transportation, and even the ability to receive postal notification of healthcare appointments when not living in stable housing [81]. The ability to engage with healthcare providers is crucial as untreated VTE has significant consequences, and chronic complications of VTE are a common cause of emergency readmissions to hospitals among PWID [82]. Through the lens of a syndemic framework, clearly addressing the broader social determinants of thrombosis risk (and its complications/interactions with other health issues) is vital. Advocacy for affordable housing in particular, the coordination of thrombosis care with addiction care providers, decentralizing care to the community, and prioritization of access to gender-specific health services may abrogate some of the effects of the interacting risks associated with poverty, homelessness, thrombosis, and addiction [71,83].

## 3 ISTH Report: The Impact of Social, Political, and Economic Factors on Thrombosis and Bleeding Issues and Implications for Gender-Inclusive Female Health

As outlined above, several factors relating to gender-based economic, social, and political marginalization are considered to be leading social determinants of the maternal mortality rate in sub-Saharan Africa. Literacy and the ability to access education are considered to be major social determinants of maternal health. While VTE is a less common complication of pregnancy than PPH in low-to-middle-income countries (in comparison with richer nations), it remains an important potential source of morbidity and mortality and shares many of the socio-economic features of the PPH crisis. In particular, education and awareness around pregnancy-associated VTE risk are thought to be poor in these regions. Data from the preliminary survey of the “Move for Flow” health promotion program were presented by Prof Nwagha and colleagues [84], detailing the results of a cross-sectional survey of 1000 pregnant Nigerian women attending antenatal obstetric services across 5 sites. Over 80% of survey respondents reported no knowledge of a VTE risk associated with pregnancy. In keeping with other reports of socio-economic factors influencing maternal health in sub-Saharan Africa, the investigators demonstrated significant associations between knowledge of pregnancy-associated VTE risk and levels of maternal education, employment status, and level of spousal education. These data will inform the implementation plan for the “Move for Flow” campaign, which will aim to improve maternal awareness of VTE risk in these regions.

Similarly, Tootoonchian et al. [85] demonstrated an association between case identification of von Willebrand disease (VWD) and economic status in their analysis of reports of new diagnoses of VWD as reported to the Annual Global Survey of the World Federation of Hemophilia. In this report, they demonstrated that despite global initiatives to improve awareness of VWD and to promote increased access to VWD diagnostics, large gaps still exist, with large numbers of people with VWD likely remaining undiagnosed in low-income countries (which may also represent a risk factor for PPH). Interestingly, they reported that in the period 2017-2021, the rate of case identification of VWD among females was greater than that observed among males in all global regions, suggesting that some progress has been made in improving diagnosis of bleeding disorders among women and girls (which has previously been underappreciated and underestimated). Issues around menstrual poverty in general were also addressed by Lukindo et al. [86], who devised and validated an adolescent menstrual poverty questionnaire, which aims to characterize menstrual poverty across several domains, including affordability and access to menstrual hygiene products, quality of life, etc.

Bottacari et al. [87] presented data describing challenges encountered by individuals with inherited bleeding disorders and concurrent substance use disorders/mental health disorders in accessing care in residential treatment facilities/mental health treatment facilities in the United States. People with bleeding disorders are at increased risk of psychological health issues and may also be at risk of substance use disorders in cases where prolonged exposure to opioid medications for chronic pain is required. The data presented here, generated through a survey of hemophilia care providers, suggested that stigmatization of individuals with complex care requirements related to a hemophilia diagnosis limits access to residential addiction/mental health facilities. Respondents to the survey reported that over 80% of referrals to residential behavioral health treatment were rejected based on the individual’s concurrent bleeding disorder diagnosis.

In the field of VTE, a study of factors associated with outpatient treatment of low-risk PE identified race/ethnicity as a potential factor that appeared to influence clinician decision-making around selecting such patients, although this finding requires additional confirmation/exploration [88].

## 4 Future Directions

The use of the syndemic framework to study the origins of ill health has increased substantially over the last 2 decades since the SAVA syndemic was first described in the 1990s [89]. Most of the literature to date has focused on the co-occurrence and interactions between HIV/AIDs, mental health conditions, poverty, and cardiometabolic disease [30]. More recently, syndemic features have been described in other settings, including in the recent COVID-19 pandemic and in relation to rheumatic diseases [16,[90], [91], [92]]. Thrombotic and vascular diseases, in general, may represent another potential group of diseases where the application of syndemic theory may improve health outcomes. With regards to maternal mortality, improving education around PPH and addressing current gaps in care, such as in the management of maternal anemia and removing obstacles to accessing medical attention, requires prioritization. Advocacy for improving autonomy for marginalized females in general around reproductive health is crucial. With regards to the interplay between SES and thrombosis, the commonalities in the etiology of severe deprivation, substance misuse, mental health conditions, and thrombosis also lend themselves to satisfying syndemic criteria. Clearly, in order to confirm a true syndemic, specific epidemiologic criteria must be satisfied, and so the hypothesis put forward here requires additional study and confirmation [29]. Nonetheless, recognizing the importance of the social, political, and economic factors that drive disease and designing healthcare services that address these factors and that do not lay responsibility for disease emergence on individual factors alone would be of major benefit to the most marginalized people in our society.

## References

[1] Hossain M.M., Saha N., Rodela T.T., Tasnim S., Nuzhath T., Roy T.J. (2022). Global research on syndemics: a meta-knowledge analysis (2001-2020). F1000Res.

[2] Singer M., Bulled N., Ostrach B., Mendenhall E. (2017). Syndemics and the biosocial conception of health. Lancet.

[3] Marmot M., Goldblatt P., Boyce T., McNeish D., Grady M., Geddes I. (2010).

[4] Marmot M., Allen J., Boyce T., Goldblatt P., Morrison J. (2020).

[5] Rálaigh C.Ó. (2021). What’s in a name? Applying the syndemic perspective to COVID-19 in Ireland. Ir J Sociol.

[6] Thomson K., Moffat M., Arisa O., Jesurasa A., Richmond C., Odeniyi A. (2021). Socioeconomic inequalities and adverse pregnancy outcomes in the UK and Republic of Ireland: a systematic review and meta-analysis. BMJ Open.

[7] St Martin B.S., Spiegel A.M., Sie L., Leonard S.A., Seidman D., Girsen A.I. (2021). Homelessness in pregnancy: perinatal outcomes. J Perinatol.

[8] Havranek E.P., Mujahid M.S., Barr D.A., Blair I.V., Cohen M.S., Cruz-Flores S. (2015). Social determinants of risk and outcomes for cardiovascular disease: a scientific statement from the American Heart Association. Circulation.

[9] Kokosi T., Flouri E., Midouhas E. (2021). The role of inflammation in the association between poverty and working memory in childhood. Psychoneuroendocrinology.

[10] Kokosi T., Flouri E., Midouhas E. (2020). Do upsetting life events explain the relationship between low socioeconomic status and systemic inflammation in childhood? Results from a longitudinal study. Brain Behav Immun.

[11] World Health Organization Closing the gap in a generation: Health equity through action on the social determinants of health. Commission on the Social Determinants of Health 2008. https://www.who.int/publications/i/item/WHO-IER-CSDH-08.1.

[12] Powell-Wiley T.M. (2023). Centering patient voices through community engagement in cardiovascular research. Circulation.

[13] Mendenhall E. (2017). Syndemics: a new path for global health research. Lancet.

[14] Blank M.B. (2015). Homelessness, mental illness, substance abuse, and HIV: an insidious syndemic. HIV/AIDS Res Treat Open J.

[15] Al Mahmeed W., Al-Rasadi K., Banerjee Y., Ceriello A., Cosentino F., Galia M. (2021). Promoting a syndemic approach for cardiometabolic disease management during COVID-19: the CAPISCO International Expert Panel. Front Cardiovasc Med.

[16] Gravlee C.C. (2020). Systemic racism, chronic health inequities, and COVID-19: a syndemic in the making?. Am J Hum Biol.

[17] Hatcher A.M., Gibbs A., McBride R.S., Rebombo D., Khumalo M., Christofides N.J. (2022). Gendered syndemic of intimate partner violence, alcohol misuse, and HIV risk among peri-urban, heterosexual men in South Africa. Soc Sci Med.

[18] Newfield T.P. (2022). Syndemics and the history of disease: towards a new engagement. Soc Sci Med.

[19] Mendenhall E. (2012).

[20] Dirisu O., Adediran M., Omole A., Akinola A., Ebenso B., Shoyemi E. (2022). The syndemic of substance use, high-risk sexual behavior, and violence: a qualitative exploration of the intersections and implications for HIV/STI prevention among key populations in Lagos, Nigeria. Front Trop Dis.

[21] Singer M. (2013). Development, coinfection, and the syndemics of pregnancy in Sub-Saharan Africa. Infect Dis Poverty.

[22] Spector A.L., Quinn K.G., deRoon-Cassini T.A., Young S.A., O'Brien M., Dickson-Gomez J. (2022). Syndemics and the etiology of opioid misuse among women: a qualitative study. SSM Qual Res Health.

[23] Hill A.V., Mendez D.D., Haggerty C.L., Miller E., De Genna N.M. (2022). Syndemics of sexually transmitted infections in a sample of racially diverse pregnant young women. Matern Child Health J.

[24] Tsuyuki K., Chan E., Lucea M.B., Cimino A., Rudolph A.E., Tesfai Y. (2023). Characterising a syndemic among black women at risk for HIV: the role of sociostructural inequity and adverse childhood experiences. Sex Transm Infect.

[25] Chhim S., Ngin C., Chhoun P., Tuot S., Ly C., Mun P. (2017). HIV prevalence and factors associated with HIV infection among transgender women in Cambodia: results from a national integrated biological and behavioral survey. BMJ Open.

[26] Radix A., Sevelius J., Deutsch M.B. (2016). Transgender women, hormonal therapy and HIV treatment: a comprehensive review of the literature and recommendations for best practices. J Int AIDS Soc.

[27] Singer M. (1996). A dose of drugs, a touch of violence, a case of AIDS: conceptualizing the SAVA syndemic. Free Inq Creat Sociol.

[28] Martinez O., Brady K.A., Levine E., Page K.R., Zea M.C., Yamanis T.J. (2020). Using syndemics theory to examine HIV sexual risk among Latinx men who have sex with men in Philadelphia, PA: findings from the National HIV Behavioral Surveillance. EHQUIDAD.

[29] Mendenhall E., Newfield T., Tsai A.C. (2022). Syndemic theory, methods, and data. Soc Sci Med.

[30] Mendenhall E., Kohrt B.A., Norris S.A., Ndetei D., Prabhakaran D. (2017). Non-communicable disease syndemics: poverty, depression, and diabetes among low-income populations. Lancet.

[31] Tsai A.C., Mendenhall E., Trostle J.A., Kawachi I. (2017). Co-occurring epidemics, syndemics, and population health. Lancet.

[32] Aldridge R.W., Story A., Hwang S.W., Nordentoft M., Luchenski S.A., Hartwell G. (2018). Morbidity and mortality in homeless individuals, prisoners, sex workers, and individuals with substance use disorders in high-income countries: a systematic review and meta-analysis. Lancet.

[33] Lynn E., Devin J., Craig S., Lyons S. (2023).

[34] Ni Cheallaigh C., Cullivan S., Sears J., Lawlee A.M., Browne J., Kieran J. (2017). Usage of unscheduled hospital care by homeless individuals in Dublin, Ireland: a cross-sectional study. BMJ Open.

[35] World Health Organization (2023). https://www.who.int/publications/i/item/9789240068759.

[36] United Nations Populations Fund (2012). https://www.unfpa.org/resources/social-determinants-maternal-death-and-disability.

[37] World Health Organization A roadmap to combat postpartum haemorrhage between 2023 and 2030. https://www.who.int/publications/i/item/9789240081802.

[38] Batist J. (2019). An intersectional analysis of maternal mortality in Sub-Saharan Africa: a human rights issue. J Glob Health.

[39] Guttmacher Institute (2012). https://www.prb.org/resources/unmet-need-for-contraception-fact-sheet.

[40] WOMAN Trial Collaborators (2017). Effect of early tranexamic acid administration on mortality, hysterectomy, and other morbidities in women with post-partum haemorrhage (WOMAN): an international, randomised, double-blind, placebo-controlled trial. Lancet.

[41] Picetti R., Miller L., Shakur-Still H., Pepple T., Beaumont D., Balogun E. (2020). The WOMAN trial: clinical and contextual factors surrounding the deaths of 483 women following post-partum haemorrhage in developing countries. BMC Pregnancy Childbirth.

[42] Jorgensen H., Horvath-Puho E., Laugesen K., Braekkan S., Hansen J.B., Sorensen H.T. (2021). Socioeconomic status and risk of incident venous thromboembolism. J Thromb Haemost.

[43] Isma N., Merlo J., Ohlsson H., Svensson P.J., Lindblad B., Gottsater A. (2013). Socioeconomic factors and concomitant diseases are related to the risk for venous thromboembolism during long time follow-up. J Thromb Thrombolysis.

[44] Zoller B., Li X., Sundquist J., Sundquist K. (2012). Socioeconomic and occupational risk factors for venous thromboembolism in Sweden: a nationwide epidemiological study. Thromb Res.

[45] Kort D., van Rein N., van der Meer F.J.M., Vermaas H.W., Wiersma N., Cannegieter S.C. (2017). Relationship between neighborhood socioeconomic status and venous thromboembolism: results from a population-based study. J Thromb Haemost.

[46] Tawakol A., Osborne M.T., Wang Y., Hammed B., Tung B., Patrich T. (2019). Stress-associated neurobiological pathway linking socioeconomic disparities to cardiovascular disease. J Am Coll Cardiol.

[47] Hicks B., Veronesi G., Ferrario M.M., Forrest H., Whitehead M., Diderichsen F. (2021). Roles of allostatic load, lifestyle and clinical risk factors in mediating the association between education and coronary heart disease risk in Europe. J Epidemiol Community Health.

[48] Baumeister D., Akhtar R., Ciufolini S., Pariante C.M., Mondelli V. (2016). Childhood trauma and adulthood inflammation: a meta-analysis of peripheral C-reactive protein, interleukin-6 and tumour necrosis factor-alpha. Mol Psychiatry.

[49] Danese A., Moffitt T.E., Harrington H., Milne B.J., Polanczyk G., Pariante C.M. (2009). Adverse childhood experiences and adult risk factors for age-related disease: depression, inflammation, and clustering of metabolic risk markers. Arch Pediatr Adolesc Med.

[50] Farmakis I.T., Valerio L., Giannakoulas G., Hobohm L., Cushman M., Piazza G. (2023). Social determinants of health in pulmonary embolism management and outcome in hospitals: insights from the United States nationwide inpatient sample. Res Pract Thromb Haemost.

[51] Wadhera R.K., Secemsky E.A., Wang Y., Yeh R.W., Goldhaber S.Z. (2021). Association of socioeconomic disadvantage with mortality and readmissions among older adults hospitalized for pulmonary embolism in the United States. J Am Heart Assoc.

[52] Misra S., Earle W., Li S., Wester A., Secemsky E.A., Carroll B. (2023). Socioeconomic distress, race, and risk of adverse outcomes after hospitalization for pulmonary embolism. J Am Coll Cardiol.

[53] Hafeez M.S., Phillips A.R., Reitz K.M., Sridharan N.D., Avgerinos E., Chaer R.A. (2022). Socioeconomic disadvantage is associated with health care disparities in mortality and readmissions after submassive (intermediate-risk) pulmonary embolism. J Vasc Surg.

[54] Wiredu C., Haynes N., Guerra C., Ky B. (2022). Racial and ethnic disparities in cancer-associated thrombosis. Thromb Haemost.

[55] Cohen A.T., Sah J., Dhamane A.D., Lee T., Rosenblatt L., Hlavacek P. (2021). Effectiveness and safety of apixaban versus warfarin among older patients with venous thromboembolism with different demographics and socioeconomic status. Adv Ther.

[56] Jorgensen H., Horvath-Puho E., Laugesen K., Braekkan S.K., Hansen J.B., Sorensen H.T. (2022). The interaction between venous thromboembolism and socioeconomic status on the risk of disability pension. Clin Epidemiol.

[57] Jorgensen H., Horvath-Puho E., Laugesen K., Braekkan S., Hansen J.B., Sorensen H.T. (2021). Risk of a permanent work-related disability pension after incident venous thromboembolism in Denmark: a population-based cohort study. PLoS Med.

[58] O'Donnell P., O'Donovan D., Elmusharaf K. (2018). Measuring social exclusion in healthcare settings: a scoping review. Int J Equity Health.

[59] World Health Organization (2010). https://www.who.int/publications/i/item/poverty-and-social-exclusion-in-the-who-european-region.

[60] Fazel S., Geddes J.R., Kushel M. (2014). The health of homeless people in high-income countries: descriptive epidemiology, health consequences, and clinical and policy recommendations. Lancet.

[61] Doran J., Hope V., Wright T., Scott J., Ciccarone D., Harris M. (2022). Prevalence and factors associated with chronic venous insufficiency, leg ulceration and deep-vein thrombosis among people who inject drugs in London, UK. Drug Alcohol Rev.

[62] Szlaszynska M., Forgo G., Fumagalli R.M., Mazzaccaro D., Nano G., Kucher N. (2023). Venous thromboembolism and chronic venous disease among people who inject drugs: a systematic review and meta-analysis. Thromb Update.

[63] Williams K., Abbey E. (2006). Knowledge of deep vein thrombosis among intravenous drugmisusers. Psychiatr Bull.

[64] Jain N., Avanthika C., Singh A., Jhaveri S., De la Hoz I., Hassen G. (2021). Deep vein thrombosis in intravenous drug users: an invisible global health burden. Cureus.

[65] Cornford C.S., Mason J.M., Inns F. (2011). Deep vein thromboses in users of opioid drugs: incidence, prevalence, and risk factors. Br J Gen Pract.

[66] Tuchman E. (2015). Women's injection drug practices in their own words: a qualitative study. Harm Reduct J.

[67] McColl M.D., Tait R.C., Greer I.A., Walker I.D. (2001). Injecting drug use is a risk factor for deep vein thrombosis in women in Glasgow. Br J Haematol.

[68] W Batchelder A., Lounsbury D.W., Palma A., Carrico A., Pachankis J., Schoenbaum E. (2016). Importance of substance use and violence in psychosocial syndemics among women with and at-risk for HIV. AIDS Care.

[69] Nydegger L.A., Claborn K.R. (2020). Exploring patterns of substance use among highly vulnerable Black women at-risk for HIV through a syndemics framework: a qualitative study. PLoS One.

[70] Carpenter C., O' Farrell A., Ni Ainle F., Ni Cheallaigh C., Kevane B. (2024). Retrospective cross-sectional analysis of concurrent VTE diagnosis in hospitalised socially excluded individuals in Ireland. BMJ Open.

[71] Caton C.L., El-Bassel N., Gelman A., Barrow S., Herman D., Hsu E. (2013). Rates and correlates of HIV and STI infection among homeless women. AIDS Behav.

[72] Beijer U., Andreasson S. (2009). Physical diseases among homeless people: gender differences and comparisons with the general population. Scand J Public Health.

[73] Corey E., Frazin S., Heywood S., Haider S. (2020). Desire for and barriers to obtaining effective contraception among women experiencing homelessness. Contracept Reprod Med.

[74] McGeough C., Walsh A., Clyne B. (2020). Barriers and facilitators perceived by women while homeless and pregnant in accessing antenatal and or postnatal healthcare: a qualitative evidence synthesis. Health Soc Care Community.

[75] de Jong C.M.M., Blondon M., Ay C., Buchmuller A., Beyer-Westendorf J., Biechele J. (2022). Incidence and impact of anticoagulation-associated abnormal menstrual bleeding in women after venous thromboembolism. Blood.

[76] Jaafar H., Ismail S.Y., Azzeri A. (2023). Period poverty: a neglected public health issue. Korean J Fam Med.

[77] Rohatgi A., Dash S. (2023). Period poverty and mental health of menstruators during COVID-19 pandemic: lessons and implications for the future. Front Glob Womens Health.

[78] Maschiao L.F., Bastos F.I., Wilson E., McFarland W., Turner C., Pestana T. (2020). Nonprescribed sex hormone use among trans women: the complex interplay of public policies, social context, and discrimination. Transgend Health.

[79] O'Rourke E.C.C., Toolan S., Solomon L., Ní Áinle F., Kevane B. (2023). The burden of venous thromboembolism and the challenges in delivering adequate care in the setting of poverty, homelessness and social exclusion: a single centre cross-sectional study [abstract]. Hemasphere.

[80] Appelhans B.M. (2023). The cognitive burden of poverty: a mechanism of socioeconomic health disparities. Am J Prev Med.

[81] O'Carroll A., Wainwright D. (2021). Doctor-patient interactions that exclude patients experiencing homelessness from health services: an ethnographic exploration. BJGP Open.

[82] Senbanjo R., Tipping T., Hunt N., Strang J. (2012). Injecting drug use via femoral vein puncture: preliminary findings of a point-of-care ultrasound service for opioid-dependent groin injectors in treatment. Harm Reduct J.

[83] Jagpal P., Saunders K., Plahe G., Russell S., Barnes N., Lowrie R. (2020). Research priorities in healthcare of persons experiencing homelessness: outcomes of a national multi-disciplinary stakeholder discussion in the United Kingdom. Int J Equity Health.

[84] Ojukwu C., Nwagha T., Agbo L., Ezeigwe A. (2023). Knowledge of venous thromboembolism among Nigeria pregnant women: a preliminary survey for the "Move for Flow" program [abstract]. Res Pract Thromb Haemost.

[85] Tootoonchian E., Stonebraker J., Iorio A., El-Ekiaby M., Gouider E., Makris M. (2023). Analysis of the change in diagnosis of von Willebrand disease by region and economic status [abstract]. Res Pract Thromb Haemost.

[86] Lukindo M., Cameron H., Pike M., Bouchard M., Price V. (2023). Validation of the English adolescent menstrual poverty questionnaire (aMPQ) [abstract]. Res Pract Thromb Haemost.

[87] Bottacari J., Feinstein M., Feldman J., Goldstein M., Hobraczk M., Mann Z. (2023). Equitable care for individuals with inherited bleeding and substance use disorders [abstract]. Res Pract Thromb Haemost.

[88] Chang K., Wang G., Giordano N., Kabrhel C. (2023). Factors associated with outpatient treatment among patients with low-risk pulmonary embolism [abstract]. Res Pract Thromb Haemost.

[89] Dixon J., Mendenhall E. (2023). Syndemic thinking to address multimorbidity and its structural determinants. Nat Rev Dis Primers.

[90] Gilcrease G.W., Sciascia S., Padovan D., Sciullo A., Cioffi M., Ricceri F. (2023). Health inequalities and social determinants of health: the role of syndemics in rheumatic diseases. Autoimmun Rev.

[91] Wildman J.M., Morris S., Pollard T., Gibson K., Moffatt S. (2022). "I wouldn't survive it, as simple as that": syndemic vulnerability among people living with chronic non-communicable disease during the COVID-19 pandemic. SSM Qual Res Health.

[92] Kapoor N., Kalra S., Al Mahmeed W., Al-Rasadi K., Al-Alawi K., Banach M. (2022). The dual pandemics of COVID-19 and obesity: bidirectional impact. Diabetes Ther.

